# Diverse Interleukin-7 mRNA Transcripts in Chinese Tree Shrew (*Tupaia belangeri chinensis*)

**DOI:** 10.1371/journal.pone.0099859

**Published:** 2014-06-19

**Authors:** Dandan Yu, Ling Xu, Xiao-Hong Liu, Yu Fan, Long-Bao Lü, Yong-Gang Yao

**Affiliations:** 1 Key Laboratory of Animal Models and Human Disease Mechanisms of Chinese Academy of Sciences & Yunnan Province, Kunming Institute of Zoology, Kunming, Yunnan, China; 2 Kunming College of Life Science, University of Chinese Academy of Sciences, Kunming, Yunnan, China; 3 Experimental Animal Core Facility and Kunming Primate Research Center, Kunming Institute of Zoology, Chinese Academy of Sciences, Kunming, China; University of Texas Health Science Center at San Antonio, United States of America

## Abstract

Interleukin-7 (IL7) is a pleiotropic cytokine that is actively involved in the immune system. The Chinese tree shrew (*Tupaia belangeri chinensis*) has been proposed as an alternative experimental animal to primates in biomedical research. However, there is a lack of biological knowledge about the immune system of the tree shrew. In this study, we cloned the *IL7* gene (*tIL7*) in the Chinese tree shrew and quantified the expression of mRNA transcripts in eight tissues (heart, liver, spleen, lung, kidney, intestine, skeletal muscle and brain) from 20 individuals. Eleven *tIL7* mRNA transcripts were identified in different tissues. The canonical form (*tIL7c*) had a length of 1817 bp and encoded a predicted gene product with 177 amino acids. Phylogenetic analyses based on the amino acid sequences revealed a considerably large genetic difference between tree shrew and human. Quantification of mRNA expression of transcripts *tIL7c*, *tIL7-sv1*, *tIL7-sv2* and *tIL7-sv3* showed that these transcripts were expressed in all tissues, albeit the expression levels varied in different tissues. Transcripts *tIL7c*, *tIL7-sv1*, and *tIL7-sv2* had the lowest expression in brain, and *tIL7-sv3* had a dramatically high mRNA expression in skeletal muscle and heart. The mRNA expression levels of *tIL7c* and *tIL7-sv1* were significantly increased upon ploy(I:C) stimulation in tree shrew primary renal cells. As with human full-length IL7, tIL7c, tIL7-sv1, tIL7-sv2 and tIL7-sv3 showed similar a subcellular localization pattern. Our results identified diverse *tIL7* transcripts in the Chinese tree shrew, which may play a potential role in modulating IL7-regulated biological effects.

## Introduction

Interleukin-7 (IL7) was first characterized in human as a growth factor of B lineage cells [1], while now it is a well-known multifunctional cytokine. IL7 plays an active role in the development, survival, maintaining and restoring homeostasis of mature T lymphocytes [2], [3]. It is also a key regulator of the commitment, survival, proliferation and maturation of B cells during development [4]. Furthermore, IL7 can improve the antiviral function and expansion of natural killer (NK) cells [5], [6] and regulate the development and differentiation of dendritic cells [7]. IL7 is produced by stromal cells in bone marrow and thymus [8], [9] as well as other types of cell, such as keratinocytes [10], hepatocytes [11], and epithelial cells [12]. Besides its pleiotropic role in the immune system, IL7 has been reported as a regulator of the development of central nervous system [13] and myogenesis and skeletal muscle cell migration [14].

Tree shrews (*Tupaia belangeri*) are squirrel-like animals inhabiting in the tropical shrubs or forests of South and Southeast Asia [15], as well as South China [16]. It has the highest brain-to-body mass ratio of known mammals. Because tree shrews share some characteristics of primates and insectivores, the exact taxonomic position of tree shrew has been on debate [17]–[20]. The viewpoint that tree shrew has a close affinity with primates has been recently supported by genome sequencing of a Chinese tree shrew and comparison with 14 other species [20]. Due to these unique characteristics of experimental animals, such as small body size, short reproductive cycle and life span, and low-cost of maintenance, tree shrew has been proposed to be an alternative experimental animal to primates in biomedical research [16]. Indeed, there are some spontaneous diseases, e.g. diabetes and tumor, in captured tree shrews [21], [22]. So far, tree shrew has been reported to be susceptible to infection with a wide range of human pathogenic viruses [23], including HBV [24]–[26], HCV [27], and HSV [28]. However, there are still many obstacles, especially low efficiency of infection and unknown mechanism, which disabled our attempts to establish a repeatable and stable tree shrew model for these human viruses. To collect more basic knowledge about the immune system and important genes that are related to pathogen infection and surveillance in tree shrew will undoubtedly pave the way to fulfill our ambitious task.

In this study, *IL7* and its mRNA transcripts were characterized in Chinese tree shrew. We analyzed their expression pattern in eight tissues of adult Chinese tree shrews and evaluated expression levels in tree shrew primary renal cells in response to poly(I:C) of different lengths. In addition, subcellular localization of overexpressed IL7 isoforms was also investigated. Our results provide valuable information on understanding the key regulator IL7 in Chinese tree shrew.

## Materials and Methods

### Experimental Animals and Ethics Statement

Chinese tree shrews were introduced from the experimental animal core facility of the Kunming Institute of Zoology, Chinese Academy of Sciences. After lethally anesthetized by diethyl ether, we collected eight different tissues, including heart, liver, spleen, lung, kidney, intestine, skeletal muscle and brain. Tissue samples were quickly dissected, immediately frozen in liquid nitrogen and were stored at −80°C. All efforts were made to minimize the suffering of animals.

The study protocol was reviewed and approved by the Institutional Animal Care and Use Committee of Kunming Institute of Zoology, Chinese Academy of Sciences.

### Total RNA Extraction and Reverse-Transcription (RT)

Total RNA was extracted from eight tissues and primary renal cells of Chinese tree shrews using RNAsimple Total RNA Kit (TIANGEN, Beijing) according to the manufacturer’s instruction. The A260/A280 ratio of total RNA was measured on a biophotometer (Eppendorf, Germany) and only these samples with a value of 1.8–2.0 were used for subsequent reverse-transcription. We also evaluated the quality and integrity of RNA samples based on the 28S and 18S rRNA bands on a 1% agarose gel. Around 2 µg total RNA was used to synthesize cDNA by using oligo-dT_18_ primer and M-MLV reverse transcriptase (Promega, USA).

### tIL7 Transcripts Cloning

**Figure 4:**
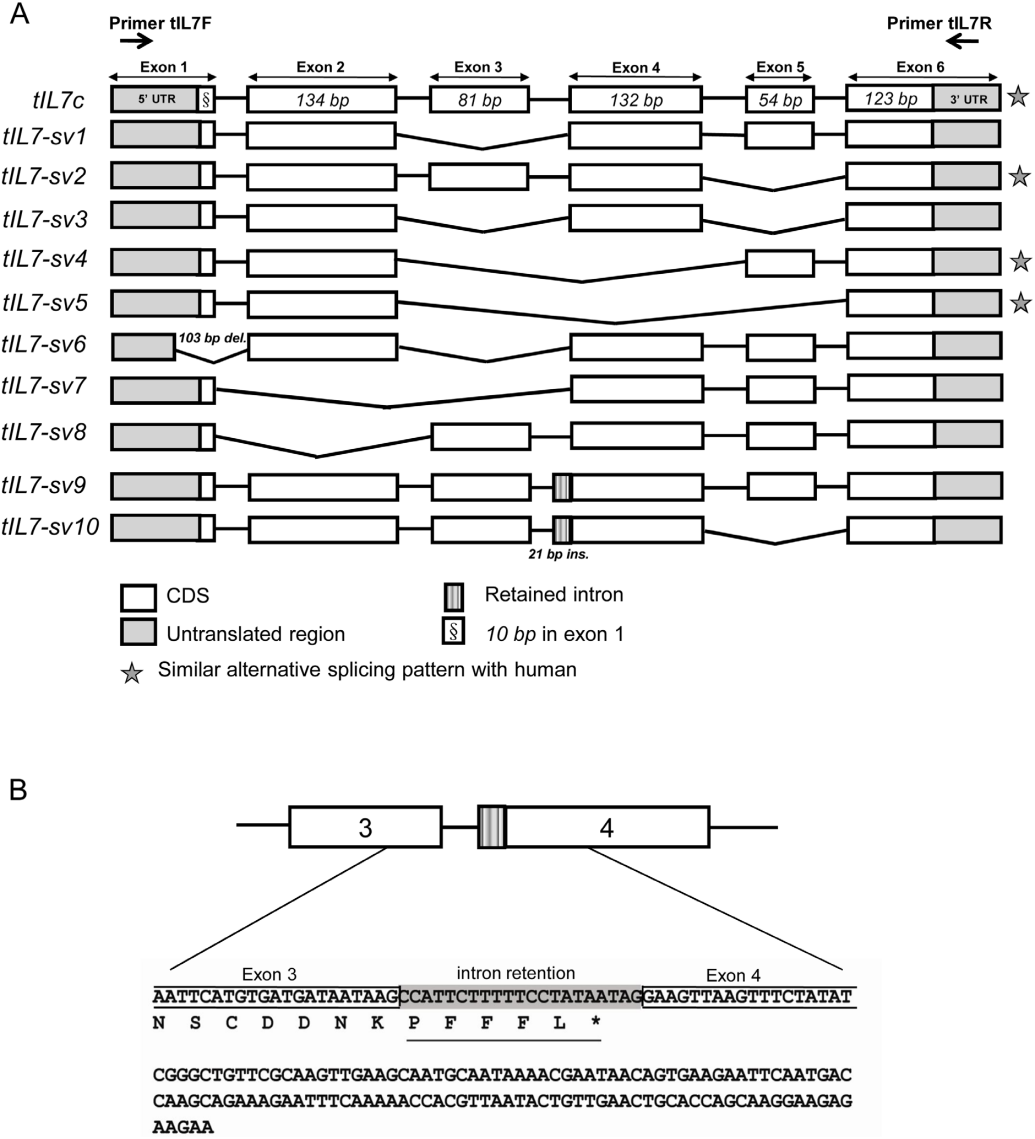
Schematic structure of *IL7* mRNA and its transcripts in Chinese tree shrew. (A) Eleven mRNA transcripts of *tIL7* gene. All transcripts were amplified by using primer pair tIL7F and tIL7R. Exons were indicated as boxes. Broken lines indicated alternative splicing of exons in *tIL7* transcripts. (B) A 21-bp insertion between exons 3 and 4 in transcripts *tIL7-sv9* and *tIL7-sv10* would result in a truncated peptide in the C-terminal of predicted protein. Transcripts *tIL7-sv6*, *tIL7-sv9* and *tIL7-sv10* complied with the splicing rule and were GT-AG introns.

Based on the predicted IL7 sequences of tree shrew retrieved from the Ensembl (http://www.ensembl.org/index.html) and the genome information of Chinese tree shrew [20] which is available at the tree shrew database (http://www.treeshrewdb.org/), a pair of primers tIL7-F and tIL7-R (Fig. 4A and Table 1) was designed to amplify the entire *IL7* gene sequence. About 1 µL cDNA synthesized from total RNA (from spleen) or pooled RNA (from all eight tissues) was used as the template. The reaction was performed in a volume of 20 µL containing 0.4 µM of each primer, 200 µM dNTPs, 1U of LA Taq DNA polymerase (TaKaRa, Dalian, China) and 2 µL 10×Buffer. We used the following PCR conditions: one denaturation cycle at 95°C for 2 min, 35 cycles of 94°C for 30 s, 55°C for 30s and 72°C for 30 s, followed by one cycle of 72°C for 5min. Purified PCR products were cloned into the PMD 19-T simple vector (TaKaRa, Dalian) and we picked up 230 positive clones for sequencing. All clones were sequenced on an automated sequencer (ABI PRISM 3730XL, Applied Biosystems) at the Kunming Biodiversity Large-Apparatus Regional Center, Kunming Institute of Zoology.

**Table 1:** Primers for cloning and quantification of *IL7* transcripts in Chinese tree shrew.

Primer	Sequence (5′-3′)	Application
*For Chinese tree shrew*		
tIL-7F	GCCGTGGACATATTAGCAAC	PCR for cloning *tIL7*
tIL-7R	ATCAAATAGCTTCAGCGTTCAG	PCR for cloning *tIL7*
tIL7 F674	CTCCCCTGATCCTTGTTCTGTTG	3′ RACE
tIL7 GSP-R1	CCCTGTTCTTACGAGGAGTTGCCTGGAG	5′ RACE
tIL7 F1239	CAGTTTTGGGGAGCAGAGTG	3′ RACE nested PCR
tIL7 GSP-R2	CAAGGGGGGCGGCACACACCAC	5′ RACE nested PCR
tIL7 SSP-F3	TGCATTGGAAGTTAAGTTTCTA	qRT-PCR for *tIL7-sv1*, *tIL7-sv3*
tIL7 SSP-F4	AAAATTCATGTGATGATAATAA	qRT-PCR for *tIL7c*
tIL7 SSP-F7	CATGTGATGATAATAAGGAAGTT	qRT-PCR for *tIL7-sv2*
tIL7 SSP-R1	TTCTTCTCTTCCTTGCTGGTG	qRT-PCR for *tIL7-sv2*, *tIL7-sv3*
tIL7 SSP-R5	TCTTTGTAGGTTGGACTTTATG	qRT-PCR for *tIL7-sv1*, *tIL7c*
tβ-actin F	ATTTTGAATGATCAGCCACC	qRT-PCR for *β-actin*
tβ-actin R	AGGTAAGCCCTGGCTGCCTC	qRT-PCR for *β-actin*
tIL7Fe1	CCGCTCGAGATGTTCCATGTTTCTTTC a	PCR for plasmids construction
tIL7Fe2	CCGCTCGAGATGGATTGTGATATTGACGGT	PCR for plasmids construction
tIL7Re2	CGCGGATCCCGTGTTTTTTAGCACCTCTC	PCR for plasmids construction
*For human*		
hIL7F	CCGCTCGAGATGTTCCATGTTTCTTTTAGGT	PCR for plasmids construction
hIL7F2	CCGCTCGAGATGGATTGTGATATTGAAGGTA	PCR for plasmids construction
hIL7R2	CGCGGATCCCGTGTTCTTTAGTGCCCATCAA	PCR for plasmids construction

a Restriction endonuclease sites introduced by PCR are underlined. RT-qPCR, quantitative real-time PCR.

In order to get a relatively intact mRNA sequence, rapid amplification of cDNA ends (RACE) was used to amplify the 5′-UTR and 3′-UTR using the SMARTer RACE cDNA Amplification Kit (Clontech, USA) and 3′ Full RACE Core Set Ver.2.0 (TaKaRa, Japan), respectively. The 5′ and 3′ RACE products were amplified using primers listed in Table 1. Purified PCR products were cloned into the PMD 19-T simple vector (TaKaRa, Dalian). Five positive clones of each insert were directly sequenced.

### Reverse Transcription Quantitative Real-Time PCR (RT-qPCR)

In order to investigate mRNA expression profile of *tIL7* and its alternative splicing transcripts in tissues and cells, transcript-specific primer pairs were designed (Table 1) and RT-qPCR was performed using SYBR green Premix Ex Taq II (TaKaRa, Dalian) on an MyIQ2 Two-Color Real-Time PCR Detection system (Bio-Rad, USA). In brief, a volume of 20 µL containing 0.4 µM of each forward and reverse primer, 1 µL of cDNA product, and 10 µL of 2×SYBR green Premix Ex Taq II were used for the RT-qPCR reaction. The tree shrew housekeeping gene β-actin was used as the reference gene for normalization. The cycling condition consisted of an initial denaturation cycle for 3 min at 95°C, 35 cycles of 30 s at 94°C, 40 s at 55°C, and a final extension step at 72°C for 15 s. In order to verify no non-specific amplification, following the completion of RT-qPCR, melting curve analysis was performed. The melting protocol consisted of heating from 55 to 95°C at a rate of 0.5°C per step, and each step was held for 1 s for data acquisition. Standard curves were generated using 10^−3^–10^−10^ dilution series of PCR product for each of the *tIL7* transcripts and β-actin gene.

### Plasmids Construction

The CDS regions of four *tIL7* transcripts (*tIL7c*, *tIL7-sv1*, *tIL7-sv2* and *tIL7-sv3*) were amplified by two primer pairs to introduce restriction endonuclease sites (*Xho* I and *BamH* I) and to cover signal peptide region. PCR products were cloned into pEGFP-N2 (Clontech, USA; Primer pair: tIL7Fe1 and tIL7Re2), respectively. Another primer pair, tIL7Fe2 and tIL7Re2 (Table 1), was designed to amplify the *tIL7* transcripts without signal peptide, and PCR fragments were inserted into pEGFP-N2. The CDS regions of human *IL7* gene (*hIL7*) with (Primer pair: hILF and hILR2) and without (Primer pair: hILF2 and hILR2) signal peptide were also cloned into pEGFP-N2 (Table 1). All constructs were verified by sequencing.

### Cell Transfection and Immunofluorescence

HeLa cell was bought from the Kunming Cell Bank, Kunming Institute of Zoology, which was initially introduced from ATCC. Cells were cultured in (Invitrogen, USA) at 37°C in 5% CO2. In brief, cells (1×10^4^ per well) were seeded in 12-well plate with coverslips and grown to 50% confluence. For each well, a total volume of 50 µL mixture containing 1 µg EGFP-tagged plasmid DNA and 2.5 µL FuGENE HD Transfection Reagent (Roche, USA) was incubated at room temperature for 20 min. Meanwhile, culture medium was removed and washed once with the OPTI-MEM medium (Invitrogen, USA). DNA/FuGENE HD complex was added to each well, together with an additional 450 µL Opti-MEM. After an incubation for 6 h, 1 mL of growth medium was added to each well. 48 h after transfection, cells were fixed with 4% paraformaldehyde for 10 min. Nuclei were stained with DAPI (Roche, USA). Subcellular localization of tIL7-EGFP were visualized by using an Olympus FluoView 1000 confocal microscope (Olympus, Melville, NY, USA).

### Isolation and Culture of Tree Shrew Primary Renal Cells and Poly(I:C) Transfection

Primary renal cells were established from 3 Chinese tree shrews with age range from 1 to 4 months. Briefly, tree shrew was sacrificed and a pair of renal was dissected. The intact renal was minced into small pieces (about 1 mm^3^) in cold PBS, and the pieces were transferred into a 50 mL sterile plastic tube containing a 1 mg/mL DNAse (Sigma, USA) and 5 mg/mL collagenase Type IV (Invitrogen, USA) solution for 30 min in 37°C water bath. After digestion, the solution was filtered through a 200-mesh sieve to remove tissue pieces. The primary renal cells were suspended and washed three times with cold PBS. Finally, cells were re-suspended and cultured at a density of 2×10^6^ cells/mL in high glucose DMEM medium supplemented with 10% FBS and 1× penicillin/streptomycin (Invitrogen, USA) at 37°C in 5% CO_2_ until confluent. For stimulation with poly(I:C), primary renal cells seeded in 12-well plates (5×10^5^ cells/well) were transfected with short or long poly(I:C) (InvivoGen, USA) at a concentration of 1 µg/mL for 6, 12 and 24 h using Lipofectamine 2000 (Invitrogen, USA) following the manufacturer’s instruction.

### Phylogenetic Analysis

To infer the phylogenetic position of Chinese tree shrew based on the *IL7* gene sequences, we retrieved *IL7* mRNA sequences of 16 species from GenBank and/or Ensembl (Table S1). Both the coding DNA sequences (CDS) and amino acid sequences were used for phylogenetic analyses. The puffer fish fugu (*Takifugu rubripes*) was used as the outgroup to root the phylogenetic tree. Trees were reconstructed using the neighbor-joining (NJ) method, maximum likelihood (ML), and minimum evolution (ME) by MEGA5.0 [29]. Since protein sequences used for phylogenetic analysis are shorter than 200 amino acid residues, we chose the Kimura 2-parameter and Poisson as the models for nucleotide sequences and amino acid sequences, respectively. Accuracies and statistical tests of phylogenetic trees were measured by bootstrap method with 1000 replications. MrBayes 3.1.2 [30], [31], which implements a Poisson model with Markov chain Monte Carlo method, was also used to obtain a phylogenetic tree. The run started with one cold chain and three heated chains for 2 million generations and every 100 sample was retained to get the final consensus tree.

### Statistical Analysis

For measurement of expression pattern of *tIL7* mRNA and its transcripts in primary renal cells with and without poly(I:C) stimulation, each assay was independently performed three times to validate the consistency of results. Data were presented as mean ± SD of three independent tests. Statistical analysis was performed using GraphPad software (GraphPad Software, La Jolla, CA, USA) with unpaired Student’s *t*-test.

## Results

### Tree Shrew IL7 Cdna Sequence and Its Amino-Acid Sequence

**Figure 1:**
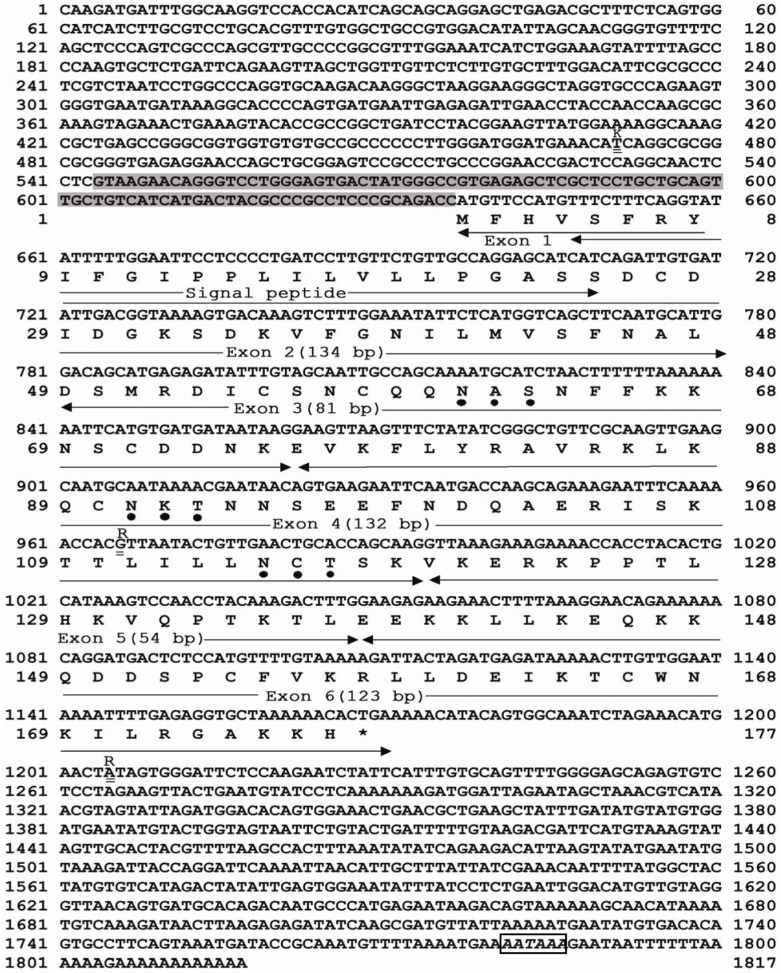
Nucleotide and deduced amino acid sequence of the *IL7* gene in Chinese tree shrew. The six exons were marked by arrows and alternative splicing fragment of transcript *tIL7-sv6* in the 5′-UTR was shaded. Potential polyadenylation signal AATAAA was marked with a box. Three predicted N-glycosylation sites were marked with dots below the respective amino acid. Three single nucleotide polymorphisms were underlined in this gene and were marked by “ = ”.

According to the predicted sequence information of tree shrew’s *IL7* gene in Ensembl and the Chinese tree shrew genome sequence generated by our own [20], we inferred that the *tIL7* gene is consisted of 6 exons. Our sequencing data showed that the full-length of *tIL7* transcript (*tIL7c*) is 1817 bp, with a 636 bp 5′-UTR, and a 647 bp 3′-UTR (including a poly-A tail) (Fig. 1). A potential polyadenylation signal AATAAA was located at 18 bp upstream of the poly-A sequence. The open reading frame (ORF) consisted of 534 nucleotides and encoded a putative polypeptide of 177 amino acid residues (Fig. 1). In the deduced gene product of tIL7, there is a signal peptide with 25 amino acid residues in the N-terminal (Fig. 1). Three potential N-glycosylation sites Asn^61^-Ala-Ser (N^61^AS), Asn^91^-Lys-Thr (N^91^KT) and Asn^115^-Cys-Thr (N^115^CT) were predicted and located in the central part according to information provided by the CBS web-server (http://www.cbs.dtu.dk/services/) (Fig. 1).

### Evolutionary Analysis of the tIL7 Gene

**Figure 2:**
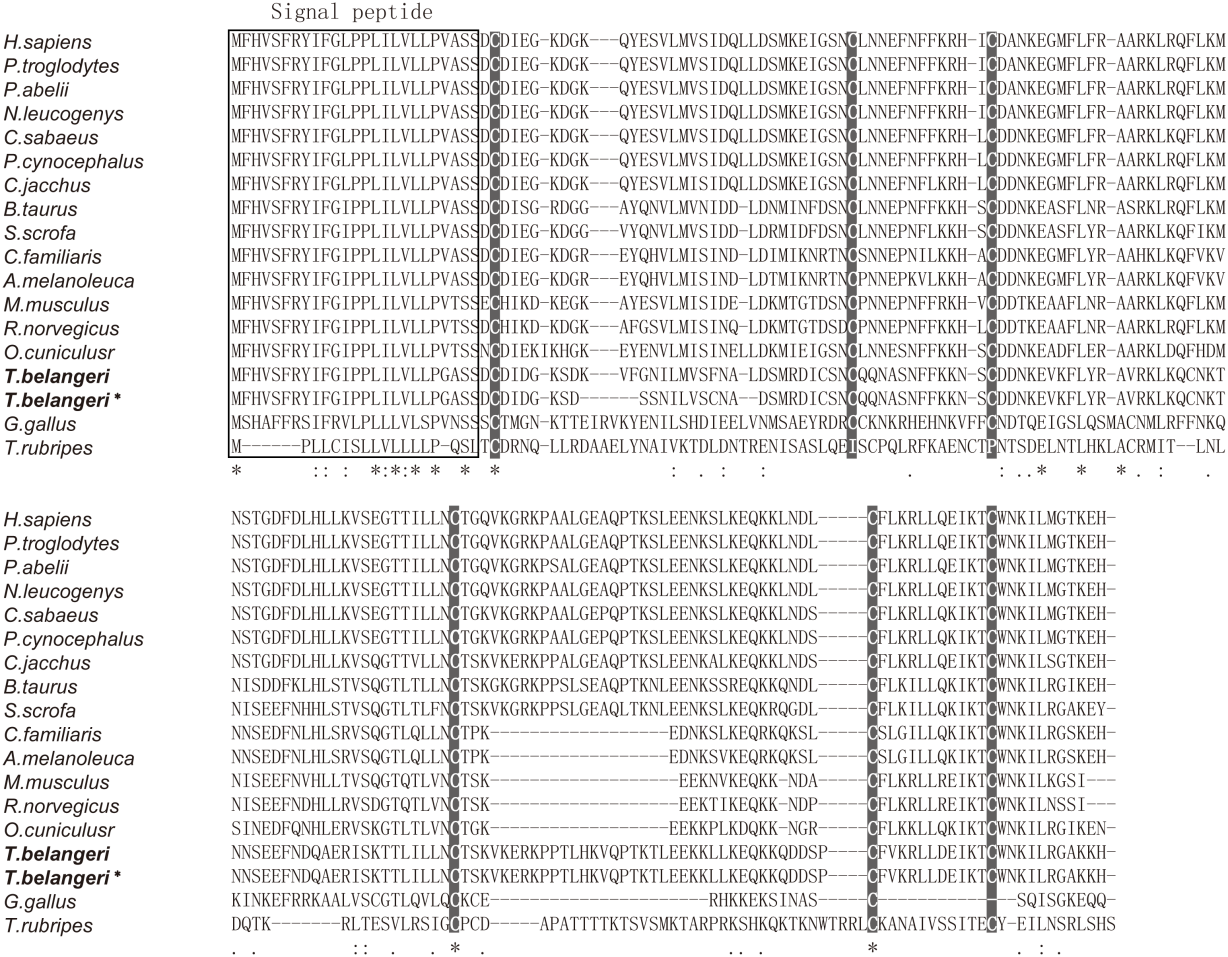
Alignment of IL7 amino acid sequences in 17 vertebrate species. The six conserved cysteine residues were marked by dark gray. “*” indicated that all residues in that column were identical in all sequences. Conserved substitutions were marked by “:”. Semi-conserved substitutions were marked by “.”. Signal peptide region was marked in box. Sequence ID information was presented in Table S1.

The Chinese tree shrew *IL7c* transcript had a considerably high nucleotide identity with human *IL7* gene (80.2%) (Table S2). In order to evaluate the evolutionary conservation of IL7 protein, sequences of multiple species were aligned together. The most conserved region was the signal peptide, in which 24 out of 25 amino acids were identical among the analyzed species except for chicken (*Gallus gallus*) (Fig. 2). In general, IL7 appeared to be a protein only highly conserved in lineage-specific species, such as primates. However, some amino acid residues were highly conserved (e.g. six cysteine residues) in all analyzed mammals (Fig. 2). These six cysteine residues in human IL7 protein can form 3 disulfides and have been reported to be important for the stability of protein three dimensional structure [32].

**Figure 3:**
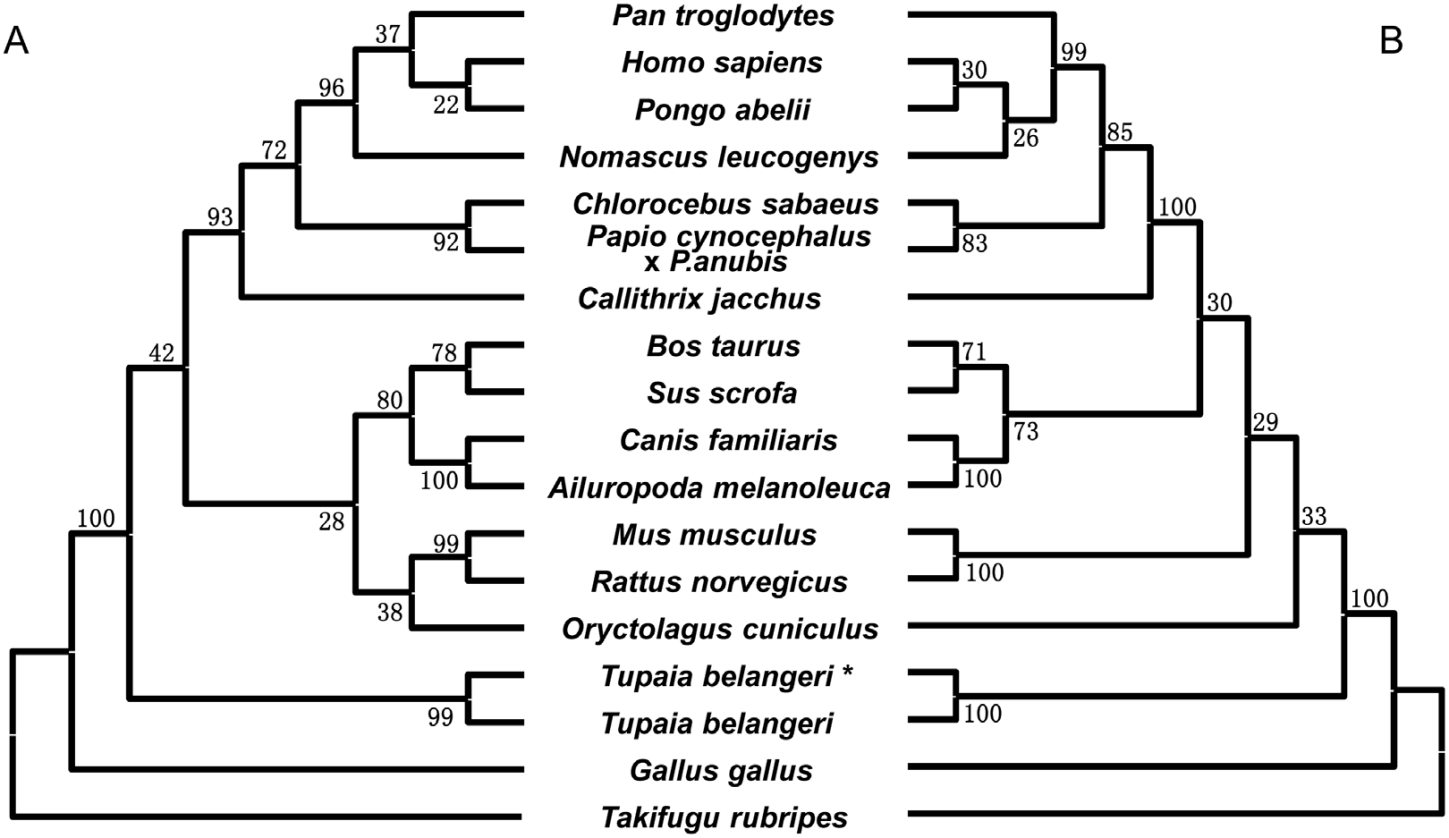
Phylogenetic trees of the *IL7* gene based on nucleotide sequences (A) and deduced protein sequences (B). The trees were reconstructed using the neighbor-joining method under the complete deletion option, with 1000 bootstrap replications. Sequence ID information was presented in Table S1. The *IL7* sequence of tree shrew retrieved from the Ensembl database was marked by “*”.

In the NJ trees that were reconstructed based on nucleotide sequence and amino acid sequence, we observed a clustering pattern that was inconsistent with the recognized species tree or gene tree based on whole genome information [20], [33], [34]. In particular, tree shrew showed a distant affinity to primates (Fig. 3). Considering the low conservation of *IL7* in different kinds of species, maximum likelihood (ML), minimum evolution (ME) and Bayesian approaches were also used to avoid the long-branch attraction when using the NJ approach to reconstruct the phylogenetic tree. We observed similar clustering pattern as the NJ trees (Fig. S1), suggesting that the result was robust.

### Identification of tIL7 Transcripts

To identify potential mRNA transcripts in Chinese tree shrew tissues, we performed sequencing for 230 cDNA clones from total RNA isolated from spleen tissue and pooled total RNA from eight tissues. Besides the above canonical transcript, a total of eleven different transcripts of the *tIL7* gene were recognized in Chinese tree shrew tissues (Fig. 4A, GenBank accession numbers JQ182399–JQ182408, KJ719472). Among them, seven transcripts, which were resulted from exon skipping, were identified in pooled total RNA from eight tissues (Fig. 4A), including *tIL7-sv1* (lacking exon 3 relative to *IL7c*), *tIL7-sv2* (lacking exon 5 relative to *IL7c*), *tIL7-sv3* (lacking exons 3 and 5 relative to *IL7c*), *tIL7-sv4* (lacking exons 3 and 4 relative to *IL7c*), *tIL7-sv5* (lacking exons 3, 4 and 5 relative to *IL7c*), t*IL7-sv7* (lacking exons 2 and 3 relative to *IL7c*, and *tIL7-sv8* (lacking exon 2 relative to *IL7c*). Despite the fact that *tIL7-sv1*, *tIL7-sv2*, *tIL7-sv3*, *tIL7-sv4* and *tIL7-sv5* lacked the corresponding amino acids encoded by the skipped exons, the remaining exons were joined in one ORF (Fig. 1). However, *tIL7-sv7* and *tIL7-sv8* might result in frameshift errors. In addition to the aforementioned exon skipping, an alternative use of splice sites, *tIL7-sv6*, which omitted exons 1 and 3 relative to *tIL7c*, was identified (Fig. 4A). Two more *tIL7* mRNA variants (*tIL7-sv9* and *tIL7-sv10*) were caused by intron retention. The retained segment is 21 bp, located in the 3′ end of the third intron, and this insertion causes a truncated polypeptide because of an alternative stop codon TAA (Fig. 4B). Transcripts *tIL7-sv6*, *tIL7-sv9* and *tIL7-sv10* complied with the splicing rule and were GT-AG introns. Among the analyzed 230 cDNA clones, ∼50% were *tIL7c* (Table S3). Presence of *IL7* transcripts had a tissue-specific pattern. For instance, *tIL7-sv5* and *tIL7-sv6* were not detected in RNA from the spleen tissue, but in pooled RNA from eight tissues we observed abundance for these two transcripts (Table S3).

### Expression Pattern of tIL7 mRNA and its Transcripts

**Figure 5:**
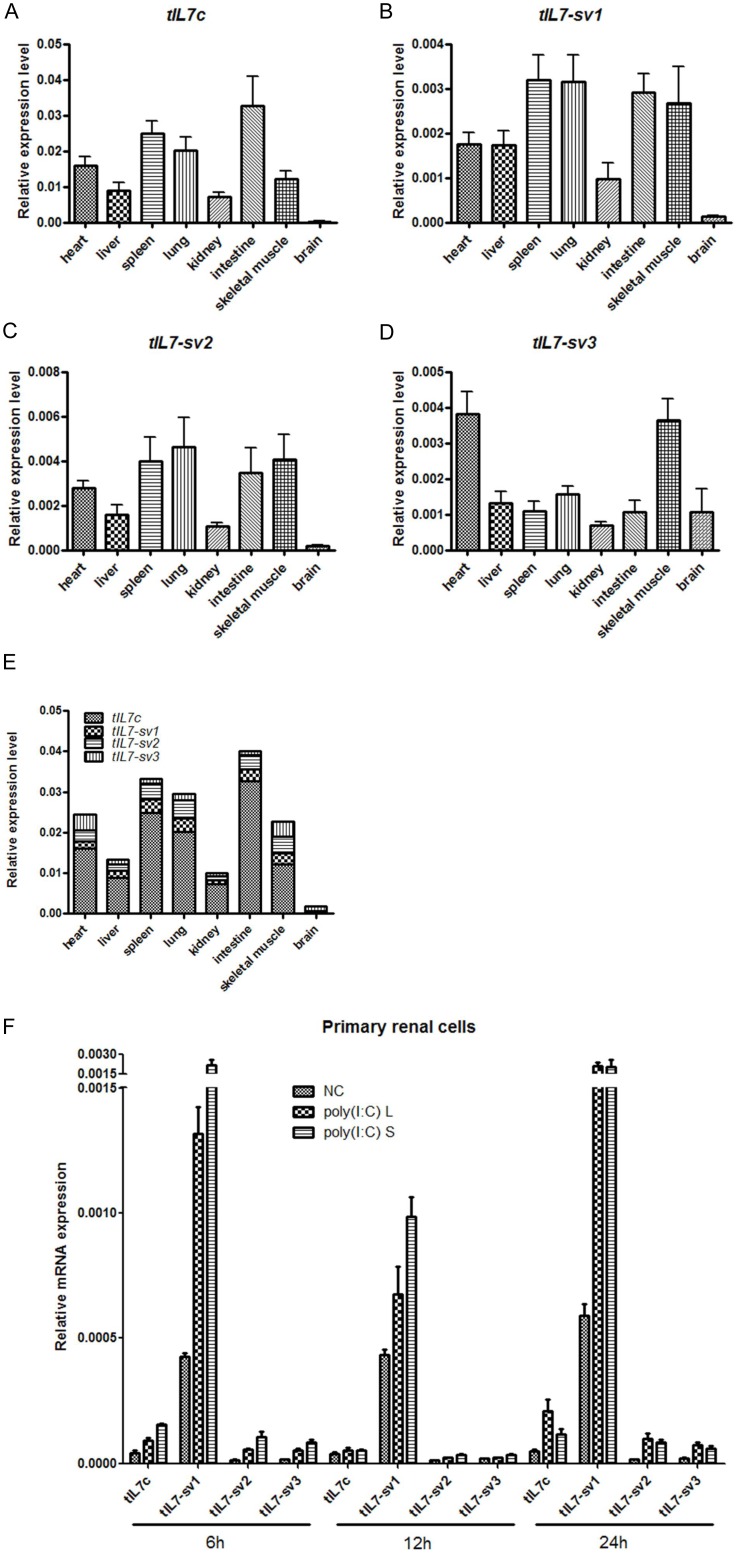
Expression patterns of *tIL7* and its transcripts in eight different tissues from 20 adult Chinese tree shrews. Relative mRNA levels of *tIL7c* (A), *tIL7-sv1* (B), *tIL7-sv2* (C), *tIL7-sv3* (D) were normalized to the amount of *β-actin* mRNA. (E) Overall expression profile of the four transcripts of *tIL7*. (F) mRNA expression of the *tIL7c* and its transcripts in primary renal cells transfected with 1 µg/mL short and long poly(I:C) at 6, 12 and 24h. NC – non-transfected cells, poly(I:C) L – long poly(I:C), poly (I:C) S – short poly(I:C). The graph shows the mean ± SD of three independent tests.

To characterize different *tIL7* transcripts, we first quantified mRNA expression profiles of *tIL7c*, *tIL7-sv1*, *tIL7-sv2*, and *tIL7-sv3* in eight different tissues from 20 adult Chinese tree shrews. These transcripts were chosen because of their relatively high abundance in above-mentioned cloning analysis. The overall expression profiles of transcripts *tIL7c*, *tIL7-sv1*, and *tIL7-sv2* were roughly same, whereas *tIL7-sv3* had a remarkable difference (Fig. 5D). Transcripts *tIL7c*, *tIL7-sv1* and *tIL7-sv2* were mainly detected in tissues related to the immune system such as intestine and spleen, as well as lung (Fig. 5A, 5B and 5C), where alveolar macrophages were widely distributed to protect the host from invading pathogens. Moderate expression levels of these three transcripts were observed in heart, skeletal muscle, liver and kidney, whereas the brain tissue had the lowest expression level (Fig. 5). The mRNA expression of transcript *tIL7-sv3* was distinguished from the other three transcripts: (1) it had a high expression level in heart and skeletal muscle rather than in tissues related to immune system; (2) brain tissue had a considerably high mRNA expression of *tIL7-sv3* (Fig. 5D). Of all analyzed tissues, *tIL7c* was the main transcript that accounted for more than 50% of all generated mRNA (excluding the brain tissue), whereas *tIL7-sv3* had an obviously prevalent expression in brain (Fig. 5E). This result is consistent with the abundance of transcripts revealed by the above cloning sequencing (Table S3).

We next assessed mRNA expression levels of *tIL7c*, *tIL7-sv1*, *tIL7-sv2* and *tIL-sv3* in primary renal cells transfected with a viral dsRNA mimic, poly(I:C), of short (0.2–1 kbp) and long (1.5–8 kbp) lengths for 6, 12 and 24h. We found that both short and long poly(I:C) obviously induced mRNA expression of *tIL7-sv1* at 6, 12 and 24 h. However, stimulation with poly(I:C) caused a trough of the *tIL7-sv1* mRNA expression at 12 h compared to 6 h and 24 h. Similar tendency was also observed for mRNA expression profile of *tIL7c*, *tIL7-sv2*, *tIL7-sv3*, and *tIL7* receptor (*tIL7R*) (Fig. 5F and Fig. S2). Short poly(I:C) had a better induction effect on these transcripts than long poly(I:C) at 6 h and 12 h, but this induction effect was similar (excluding *tIL7c*) for short poly(I:C) and long poly(I:C) at 24 h post-transfection (Fig. 5F). This pattern might reflect different reactions and signaling pathways of short and long poly(I:C) stimulation.

It should be mentioned that different expression profiles of *tIL7c* and *tIL7-sv1* transcripts were found between kidney tissue and primary renal cells. The mRNA expression level of *tIL7c* was higher than *tIL7-sv1* in kidney tissue (Fig. 5A and 5B), but *tIL7c* was lower than *tIL7-sv1* in primary renal cells (Fig. 5E and 5F). The exact reason for this discrepancy might be due to different types of cells in kidney tissue.

### Cellular Localization of tIL7 and its Transcripts

**Figure 6:**
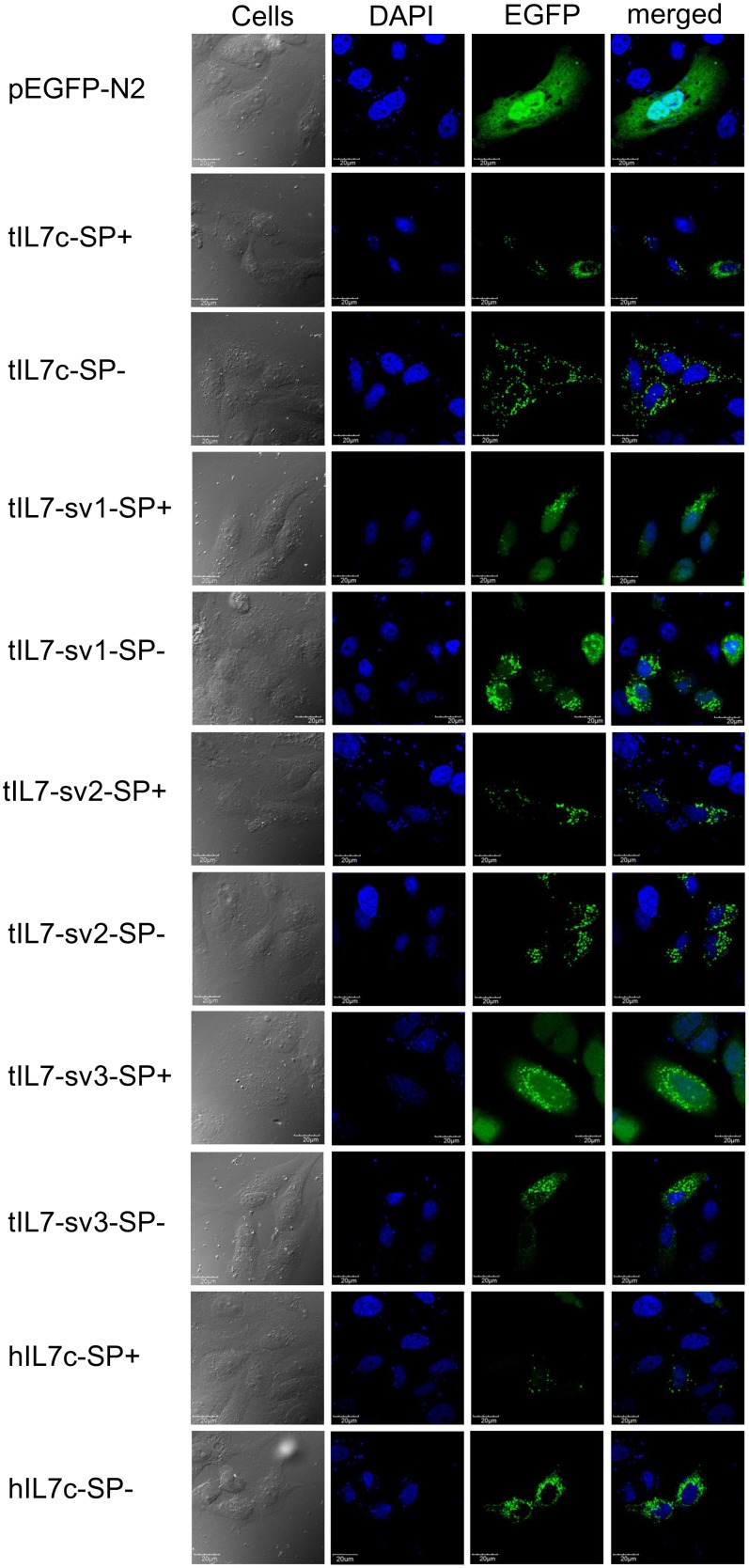
Subcellular localization of EGFP-tagged tIL7c and tIL7-sv isoforms in HeLa cells. HeLa cells were transfected with pEGFP-N2 empty vector and pEGFP-N2 vector with insert of *tIL7c* or each of the three *tIL7c* transcripts (*tIL7-sv1*, *tIL7-sv2* and *tIL7-sv3*) with (SP+) and without (SP–) the signal peptide. Immunofluorescence images were taken at 48 h after transfection. The scale marked in each section of the figure referred to 20 µm.

IL7 functions as a cytokine when released in extracellular medium. Protein function is strongly influenced by subcellular localization, and immunofluorescence microscopy was employed to determine cellular localization of the immature and mature tIL7c. EGFP-tagged tIL7c and tIL7 vectors were transfected into HeLa cells and tree shrew primary renal cells. As shown in Figure 6 and Figure S4, tIL7c without a signal peptide (tIL7c-SP-) was mainly localized to cytoplasm and presented a dot distribution, which is consistent with the pattern of hIL7-SP-. The fluorescence distribution of tIL7c-SP- was higher than the one with the signal peptide (tIL7c-SP+, Fig. 6), possibly because of the release of mature tIL7 to culture medium under the guidance of signal peptide. The other three isoforms (tIL7-sv1, tIL7-sv2, and tIL7-sv3) without a signal peptide had a similar distribution pattern with tIL7c-SP-.

## Discussion

IL7 is an important molecule in the immune system and regulates the development, differentiation, survival, and maturation of the lymphocytes [4], [35]–[37], as well as functions as antivirus, anti-tumorigenesis and anti-apoptosis for the organisms [38], [39]. Recently, *IL7* and its alternative splicing variants of human, mouse, pig, rabbit, horse, sheep, and fish were well characterized [40]–[46]. However, the existence of an *IL7* homologue in tree shrew has not been well determined so far. Because tree shrew harbored some characteristics sharing with both the ancestral and modern primates, as well as unique features as an experimental animal, it has long been proposed as an alternative animal model to primates in biomedical research [20], [23], [47]. However, lack of basic knowledge regarding the immune system genes of Chinese tree shrew has disabled our efforts to create a stable and successful animal model for infectious disease.

In this study, we cloned the *tIL7* gene and identified a total of eleven alternative splicing transcripts. The splicing region of transcripts *tIL7-sv2*, *tIL7-sv4* and *tIL7-sv5* were similar to *IL7δ5* (*hIL7* transcript lacking exon 5), *IL7δ3/4* (*hIL7* transcript lacking exons 3 and 4) and *IL7δ3/4/5* (*hIL7* transcript lacking exons 3, 4 and 5) in human, respectively. However, there are no homologues of transcripts *tIL7-sv1*, *tIL7-sv3*, *tIL7-sv6*, *tIL7-sv7*, *tIL7-sv8*, *tIL7-sv9* and *tIL7-sv10* in human, suggesting that the alternative splicing of the *IL7* gene might be different between Chinese tree shrew and human. The uniqueness of the *IL7* gene in Chinese tree shrew could be further demonstrated by the phylogenetic trees of available *IL7* gene sequences (Fig. 3), in which tree shrew showed a divergent relationship to primates.

Transcripts resulted from alternative splicing usually had tissue- and/or time-specific expression patterns and played important roles in certain tissues and/or developmental stage [40]. *tIL7c*, *tIL7-sv1*, *tIL7-sv2* and *tIL7-sv3* were highly expressed in the immune system and presented somewhat different tissue expression patterns, suggesting their active roles in immuno-regulation rather than as being nonfunctional. Human IL7 and its isoforms were reported to be regulators of central nervous system and impacted on neuronal tissue development and plasticity [13]. Moreover, IL7 and its isoforms could act as a myokine to affect myogenesis and migration [14]. The relatively high mRNA expression of *tIL7* in the heart and skeletal muscle may indicate their roles in these related systems. The distinctly high expression level of *tIL7-sv3* in brain relative to other tissues may imply a key role of this transcript in tree shrew’s central nervous system (Fig. 5). Cellular localization of these *tIL7* isoforms showed no specificity of certain isoform (Fig. 6). It should be noted that mRNA levels of these *tIL7* transcripts might not be fully correlated with protein expression levels in tissues or cells, but we do not have the necessary antibodies to recognize each tIL7 isoform.

To characterize potential function of different *tIL7* transcripts, we made several attempts, including (1) determination of mRNA expression levels of *tIL7* transcripts in primary renal cells in response to stimulation by different drug (lipopolysaccharide [LPS], poly(I:C), phytohaemagglutinin [PHA], rotenone, vitamin K3, carbonyl cyanide m-chlorophenylhydrazone [CCCP]); (2) testing for the proliferation rate of tree shrew spleen cells and primary renal cells in the presence of culture supernatant of HEK293 cells transfected with tIL7, tIL7-sv1, tIL7-sv2, or tIL7-sv3. Unfortunately, we did not obtain useful information to answer the critical question regarding the potential function of different *tIL7* transcripts. With the exception of poly(I:C) stimulation (Fig. 5F), other drugs had no apparent stimulation effect on mRNA expression levels of *tIL7* transcripts. We obtained inconsistent results regarding mRNA expression levels of *tIL7* transcripts in response to LPS treatment in renal cells from different tree shrew individuals: there was a seemingly delay of induction effect on mRNA expression of *tIL7* transcripts compared with poly(I:C) stimulation in some cells, but other cells had no response to LPS treatment (data not shown). There was no obvious difference of the proliferation rate of tree shrew spleen cells and renal cells cultured in the supernatant of HEK293 cells transfected with each of the four transcripts (tIL7, tIL7-sv1, tIL7-sv2, and tIL7-sv3) in comparison to the supernatant of HEK293 cells transfected with empty vector (Fig. S3). One potential reason for these negative observations would indicate that our system might not be optimal for distinguishing the effect of tIL7 isoforms. Several laboratories have documented the expression of IL7 in primary and secondary lymphoid organs using IL-7 reporter mice [8], [9], [48]–[50]. It may be more proper to work on thymic mesenchymal or epithelial cells. More efforts should be carried out to further define the function of these tIL7 isoforms.

Some lines of evidence showed an effect of poly(I:C) on the induction of IL7. Maternal exposure to poly(I:C) in C57BL/6J pregnant mice (gestational day 16) induced expression of IL7 in fetal mouse brain [51]. Treatment with poly(I:C) in salivary gland epithelial cells caused a significant increase of the IL7 gene expression and protein production [52]. In the Japanese pufferfish, expression of the *IL7* gene in head kidney cells increased significantly upon treatment with poly(I:C) after 4 h [44]. Concordantly, we demonstrated that poly(I:C) had an upregulation effect on mRNA expression levels of *tIL7c* and its transcripts, in particular for *tIL-sv1* (Fig. 5F). Future studies will be performed to characterize the *in vivo* effect of poly(I:C) on the induction of different IL7 isoforms and the signaling pathway underlying this upregulation effect.

In summary, we characterized expression pattern of alternative splicing variants of the *IL7* gene in Chinese tree shrew. The identification of diverse *tIL7* transcripts in Chinese tree shrew offered more food for thought: why Chinese tree shrew owns such a variety of *IL7* transcripts? What is potential function of different *tIL7* transcripts? How *tIL7* splicing is regulated during infection? How the tIL7 protein and its isoforms are modified *in vivo*? Functional study should be performed to answer these questions and to further define the regulation of alternative splicing of *tIL7* and the exact biological role of these transcripts in Chinese tree shrews.

## Supporting Information

Figure S1 **ML tree (A), ME tree (B) of IL7 amino acid sequences, with 1000 bootstrap replications and complete deletion in Gaps/Missing data.** The Bayesian tree (C) using a Poisson model with mcmc method.
(TIF)

Figure S2 **Quantitative real-time PCR analysis of the** ***tIL7R*** **gene in primary renal cells stimulated with poly(I:C) of different lengths.** Real-time PCR was performed using primer pair tIL7R F (5′-AGAATTTATCCAACACAAAACT-3′)/tIL7R R (5′-TGACCAGCAGAGCCATAGAGAG-3′) and cDNA synthesized from primary renal cells transfected with 1 µg/mL short or long poly(I:C) at 6, 12 and 24 h. The tree shrew housekeeping gene *β-actin* was used as the reference gene for normalization. NC–non-transfected cells, poly(I:C) L–long poly(I:C), poly (I:C) S–short poly(I:C). The graph shows the mean ± SD of three independent tests.
(TIF)

Figure S3 **Proliferation of tree shrew spleen cells and renal cells in response to tIL7 isoforms and hIL7. 293T cells were transfected with 10 µg each of the four transcripts (tIL7, tIL7-sv1, tIL7-sv2, and tIL7-sv3) and hIL7 or an empty vector (pcDNA3.1) in a 10 cm dish (2×10^6^ cells/dish).** Cell culture medium without FBS was replaced at 24 h post-transfection. Cells were incubated at 37°C for another 24 h, and then cell culture medium was harvested and added to tree shrew spleen cells seeded at 2×10^5^ cells/well or renal cells seeded at 2×10^4^ cells/well in 96-well plates. Proliferation of tree shrew spleen and renal cells was determined by MTT assay at 48 h. Data are presented as the mean ± SD deviation of triplicate samples.
(TIF)

Figure S4 **Subcellular localization of EGFP-tagged tIL7c and tIL7-sv isoforms in tree shrew primary renal cells.** Cells were transfected with pEGFP-N2 empty vector and pEGFP-N2 vector with insert of tIL7c or each of the three tIL7c transcripts (tIL7-sv1, tIL7-sv2 and tIL7-sv3) with (SP+) and without (SP–) the signal peptide. Immunofluorescence images were taken at 48 h after transfection. The scale marked in each section of the figure referred to 20 µm.
(TIF)

Table S1 **17 species used in the present analyses.**
(DOC)

Table S2 **Homology analysis of the** ***IL7*** **gene in 17 mammalian species.**
(DOC)

Table S3 **Percentage of clones with** ***tIL7*** **and its transcripts in mRNA isolated from tree shrew tissues.**
(DOC)
