# Supplementary figures and images for: Diverse Interleukin-7 mRNA Transcripts in Chinese Tree Shrew (Tupaia belangeri chinensis)
Source: PLoS One. 2014 Jun 19;9(6):e99859. doi: 10.1371/journal.pone.0099859 (PMC4063794; doi:10.1371/journal.pone.0099859)

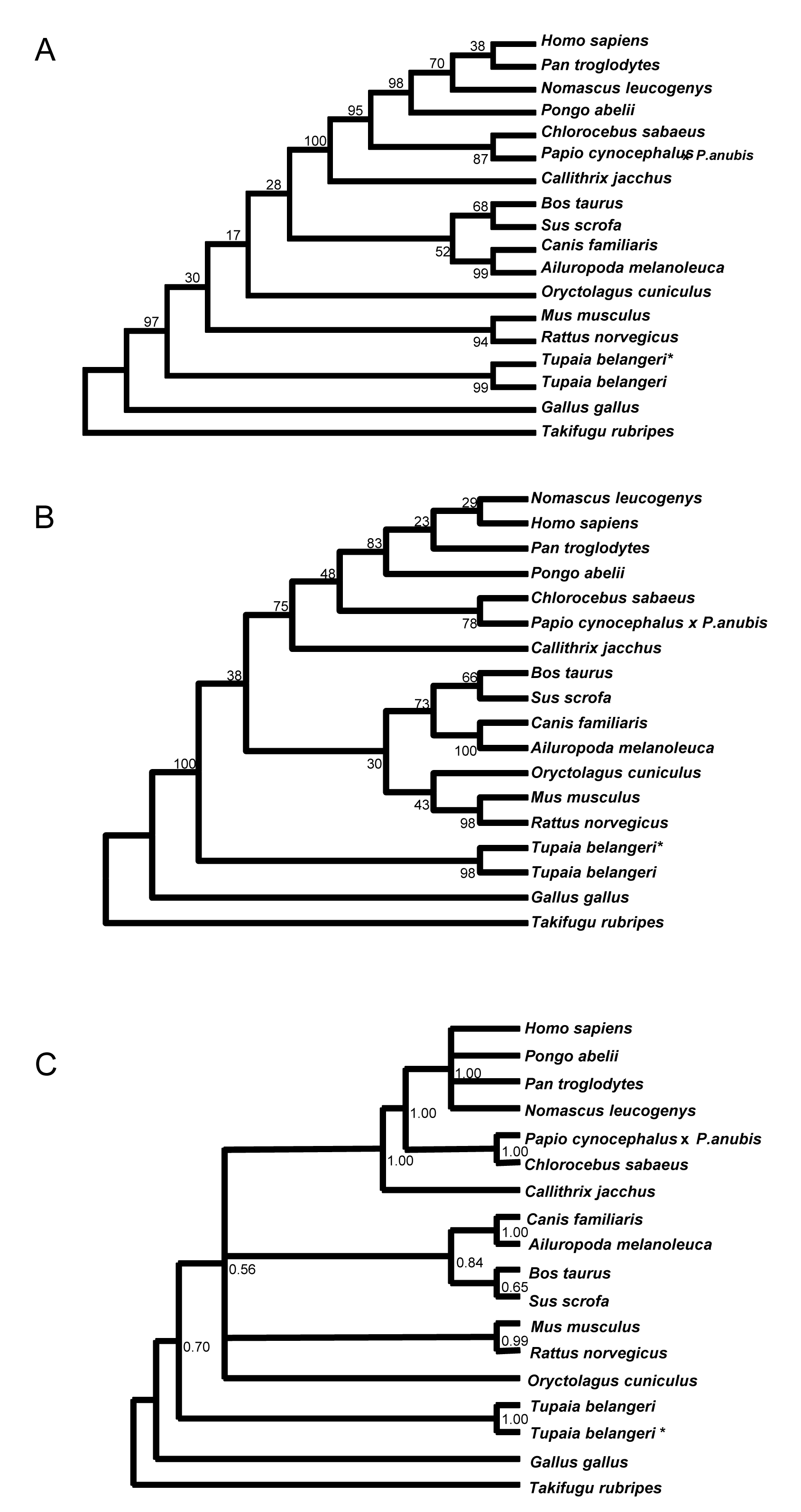

Supplement: Figure S1 — ML tree (A), ME tree (B) of IL7 amino acid sequences, with 1000 bootstrap replications and complete deletion in Gaps/Missing data. The Bayesian tree (C) using a Poisson model with mcmc method. (TIF) [file pone.0099859.s001.tif]

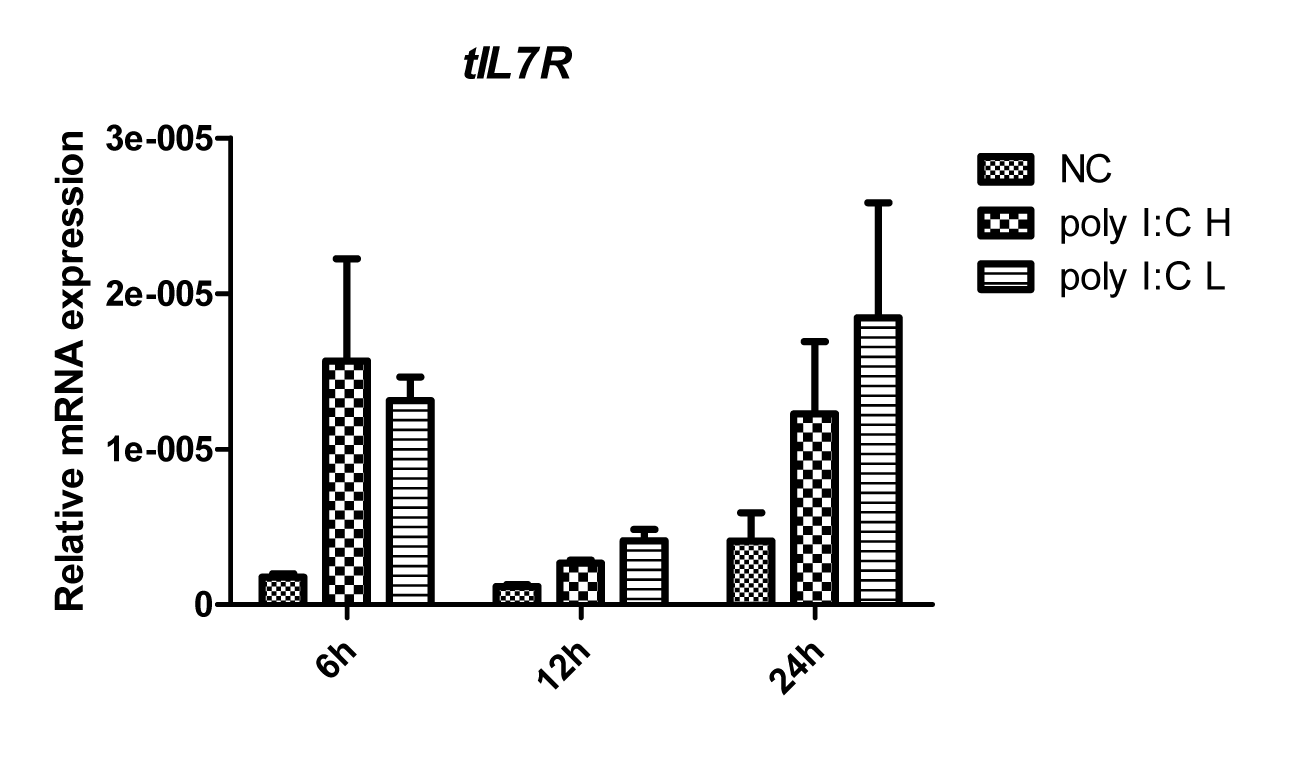

Supplement: Figure S2 — Quantitative real-time PCR analysis of the tIL7R gene in primary renal cells stimulated with poly(I:C) of different lengths. Real-time PCR was performed using primer pair tIL7R F (5′-AGAATTTATCCAACACAAAACT-3′)/tIL7R R (5′-TGACCAGCAGAGCCATAGAGAG-3′) and cDNA synthesized from primary renal cells transfected with 1 µg/mL short or long poly(I:C) at 6, 12 and 24 h. The tree shrew housekeeping gene β-actin was used as the reference gene for normalization. NC–non-transfected cells, poly(I:C) L–long poly(I:C), poly (I:C) S–short poly(I:C). The graph shows the mean ± SD of three independent tests. (TIF) [file pone.0099859.s002.tif]

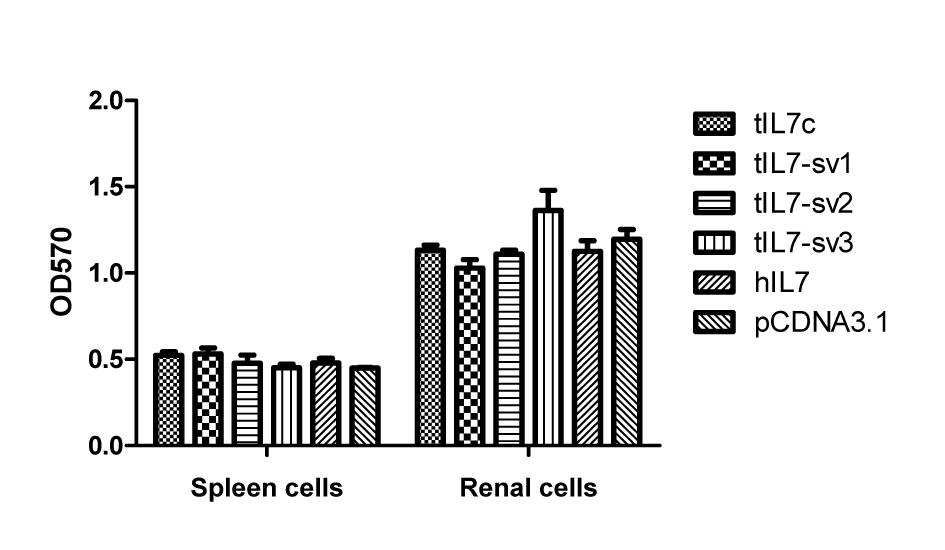

Supplement: Figure S3 — Proliferation of tree shrew spleen cells and renal cells in response to tIL7 isoforms and hIL7. 293T cells were transfected with 10 µg each of the four transcripts (tIL7, tIL7-sv1, tIL7-sv2, and tIL7-sv3) and hIL7 or an empty vector (pcDNA3.1) in a 10 cm dish (2×10^6^ cells/dish). Cell culture medium without FBS was replaced at 24 h post-transfection. Cells were incubated at 37°C for another 24 h, and then cell culture medium was harvested and added to tree shrew spleen cells seeded at 2×10^5^ cells/well or renal cells seeded at 2×10^4^ cells/well in 96-well plates. Proliferation of tree shrew spleen and renal cells was determined by MTT assay at 48 h. Data are presented as the mean ± SD deviation of triplicate samples. (TIF) [file pone.0099859.s003.tif]

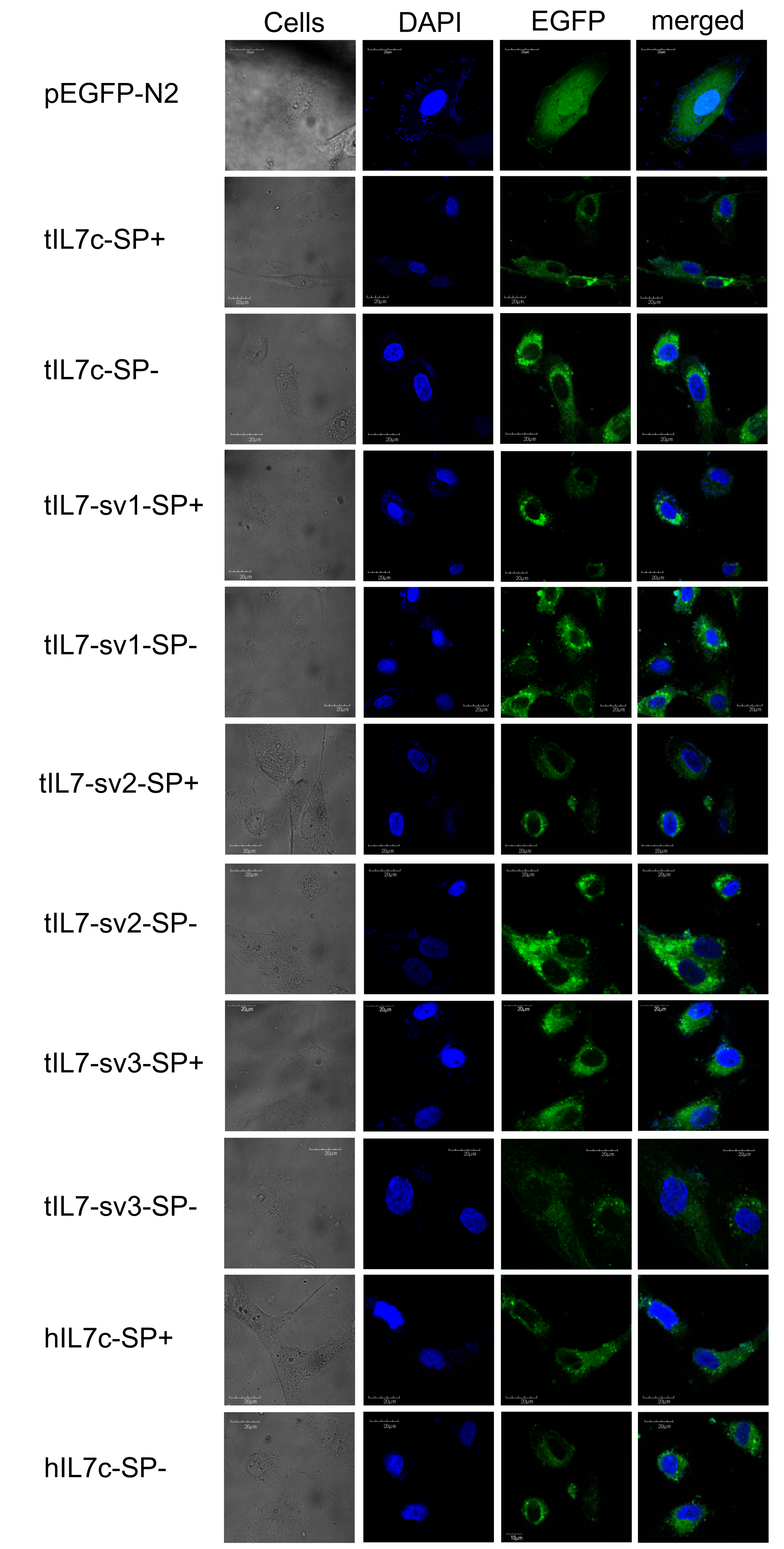

Supplement: Figure S4 — Subcellular localization of EGFP-tagged tIL7c and tIL7-sv isoforms in tree shrew primary renal cells. Cells were transfected with pEGFP-N2 empty vector and pEGFP-N2 vector with insert of tIL7c or each of the three tIL7c transcripts (tIL7-sv1, tIL7-sv2 and tIL7-sv3) with (SP+) and without (SP–) the signal peptide. Immunofluorescence images were taken at 48 h after transfection. The scale marked in each section of the figure referred to 20 µm. (TIF) [file pone.0099859.s004.tif]
