# Supplementary material for: Diverse Interleukin-7 mRNA Transcripts in Chinese Tree Shrew (Tupaia belangeri chinensis)
Source: PLoS One. 2014 Jun 19;9(6):e99859. doi: 10.1371/journal.pone.0099859 (PMC4063794; doi:10.1371/journal.pone.0099859)
Supplement: Table S1 — 17 species used in the present analyses. (DOC) [file pone.0099859.s005.doc]

Table S1. 17 species used in the present analyses

| Species | Common name | Accession number ^a^ |
| --- | --- | --- |
| *Bos taurus* | Cattle | AF348422 |
| *Gallus gallu* | Chicken | AJ852017 |
| *Papio cynocephalus* × *P.anubis* | Yellow baboon × Olive baboon | AF541946 |
| *Homo sapiens* | Human | BC047698 |
| *Canis familiaris* | Dog | DQ845341 |
| *Chlorocebus sabaeu* | Green monkey | FJ194487 |
| *Mus musculus* | House mouse | NM_008371 |
| *Rattus norvegicus* | Norway rat | NM_013110 |
| *Sus scrofa* | Pig | NM_214135 |
| *Oryctolagus cuniculus* | Rabbit | XM_002710558 |
| *Ailuropoda melanoleuca* | Giant panda | XM_002924920 |
| *Nomascus leucogenys* | Northern white-cheeked gibbon | XM_003269467 |
| *Pan troglodytes* | Chimpanzee | ENSPTRT00000037698 |
| *Callithrix jacchus* | Common marmoset | ENSCJAT00000000902 |
| *Pongo abelii* | Sumatran orangutan | ENSPPYT00000021806 |
| *Tupaia belangeri* | Northern tree shrew | ENSTBET00000007176 |
| *Takifugu rubripes* | Fugu | NM_001136148.1 |

^a^ GenBank accession number or accession number of the Ensembl data base.
