# Supplementary material for: Diverse Interleukin-7 mRNA Transcripts in Chinese Tree Shrew (Tupaia belangeri chinensis)
Source: PLoS One. 2014 Jun 19;9(6):e99859. doi: 10.1371/journal.pone.0099859 (PMC4063794; doi:10.1371/journal.pone.0099859)
Supplement: Table S2 — Homology analysis of the IL7 gene in 17 mammalian species. (DOC) [file pone.0099859.s006.doc]

Table S2. Homology analysis of the *IL7* gene in 17 mammalian species

|  | Percentage of nucleotide sequence identity | | | | | | | | | | | | | | | | | |  |
| --- | --- | --- | --- | --- | --- | --- | --- | --- | --- | --- | --- | --- | --- | --- | --- | --- | --- | --- | --- |
| Percentage of amino acid identity |  | 1 | 2 | 3 | 4 | 5 | 6 | 7 | 8 | 9 | 10 | 11 | 12 | 13 | 14 | 15 | 16 | 17 | |
|  | 1 | *** | 78.7 | 53.4 | 85.7 | 85.1 | 85.5 | 85.1 | 82.2 | 79.4 | 91.3 | 84.2 | 83.9 | 85.1 | 85.3 | 85.5 | 84.9 | 38.4 | |
|  | 2 | 63.8 | *** | 51.3 | 81.2 | **80.2** | 78 | 81 | 76.1 | 74.6 | 81 | 79.7 | 78.2 | 80.2 | 80.4 | 81.9 | 80 | 36.3 | |
|  | 3 | 31.9 | 29.1 | *** | 53.3 | 52.6 | 53.2 | 53.1 | 54.6 | 52.3 | 53.4 | 54.4 | 52.7 | 52.8 | 52.6 | 52.8 | 52.6 | 47.2 | |
|  | 4 | 75.1 | 63.8 | 31.7 | *** | 98.1 | 85.5 | 99.4 | 83 | 80.6 | 86.1 | 84.9 | 83.9 | 98.1 | 98.3 | 97 | 97.8 | 38.8 | |
|  | 5 | 74.4 | 63.1 | 31 | 96.6 | *** | 85.3 | 97.6 | 82.2 | 79.6 | 85.5 | 84.5 | 83.2 | 99.6 | 99.8 | 96.1 | 99.3 | 38.8 | |
|  | 6 | 74.8 | 62.9 | 32.1 | 71.1 | 69.6 | *** | 85.3 | 79 | 76.9 | 87.8 | 82.6 | 95.4 | 85.3 | 85.5 | 85.7 | 85.3 | 39.4 | |
|  | 7 | 74.6 | 64.4 | 31 | 99.4 | 96 | 71.7 | *** | 82.8 | 80 | 85.9 | 84.7 | 83.6 | 97.6 | 97.8 | 96.4 | 97.2 | 39 | |
|  | 8 | 72.3 | 61.9 | 31.9 | 69.7 | 69 | 64.5 | 70.3 | *** | 92.2 | 81.3 | 82.6 | 77.8 | 82.6 | 82.4 | 83.2 | 82.2 | 40 | |
|  | 9 | 71 | 65.2 | 31.2 | 70.3 | 69 | 64.5 | 69.7 | 88.4 | *** | 79.1 | 79.7 | 75.6 | 80 | 79.8 | 80.4 | 79.6 | 37.4 | |
|  | 10 | 85.9 | 68.4 | 31.2 | 74 | 73.3 | 78.6 | 74.6 | 73.5 | 71 | *** | 84 | 86.6 | 85.5 | 85.7 | 86.3 | 85.3 | 37.6 | |
|  | 11 | 72.8 | 64.6 | 33.6 | 73.6 | 71.5 | 69 | 73.6 | 71.6 | 70.3 | 74.1 | *** | 81.1 | 84.5 | 84.7 | 84.5 | 84.5 | 38.4 | |
|  | 12 | 74.8 | 62.3 | 32.1 | 70.4 | 69 | 96.2 | 71.1 | 65.8 | 65.2 | 78 | 68.4 | *** | 83.2 | 83.4 | 84.1 | 83.2 | 38.7 | |
|  | 13 | 74.6 | 63.3 | 31 | 96.6 | 100 | 69.8 | 96.1 | 69 | 69 | 73.4 | 71.7 | 69.2 | *** | 99.8 | 96.1 | 99.3 | 39 | |
|  | 14 | 74.6 | 63.3 | 31 | 96.6 | 100 | 69.8 | 96.1 | 69 | 69 | 73.4 | 71.7 | 69.2 | 100 | *** | 96.3 | 99.4 | 39 | |
|  | 15 | 74.6 | 64.4 | 31.7 | 93.8 | 92.7 | 71.7 | 93.3 | 71.6 | 71.6 | 74.6 | 72.3 | 71.1 | 92.7 | 92.7 | *** | 95.9 | 37.4 | |
|  | 16 | 74.6 | 63.3 | 31 | 96.1 | 99.4 | 69.8 | 95.5 | 69 | 69 | 73.4 | 71.7 | 69.2 | 99.4 | 99.4 | 92.7 | *** | 38.8 | |
|  | 17 | 13.8 | 13.7 | 9.4 | 11.8 | 11.8 | 12.6 | 11.8 | 14.9 | 13.5 | 13.1 | 13.2 | 11.9 | 11.8 | 11.8 | 12.4 | 11.8 | *** | |

^a^ 1 *Bos taurus*, 2 *Tupaia belangeri*, 3 *Gallus gallus*, 4 *Papio cynocephalus* × *P. anubis*, 5 *Homo sapiens*, 6 *Canis familiari*, 7 *Chlorocebus sabaeus*, 8 *Mus musculus*, 9 *Rattus norvegicus*, 10 *Sus scrofa*, 11 *Oryctolagus cuniculus*, 12 *Ailuropoda melanoleuca*, 13 *Nomascus leucogenys*, 14 *Pan troglodytes*, 15 *Callithrix jacchus*, 16 *Pongo abelii*, 17 *Takifugu rubripes*
