# Supplementary material for: Diverse Interleukin-7 mRNA Transcripts in Chinese Tree Shrew (Tupaia belangeri chinensis)
Source: PLoS One. 2014 Jun 19;9(6):e99859. doi: 10.1371/journal.pone.0099859 (PMC4063794; doi:10.1371/journal.pone.0099859)
Supplement: Table S3 — Percentage of clones with tIL7 and its transcripts in mRNA isolated from tree shrew tissues. (DOC) [file pone.0099859.s007.doc]

Table S3. Percentage of clones with *tIL7* and its transcripts in mRNA isolated from tree shrew tissues

| *Transcript* | RNA from the spleen tissue | Pooled RNA from eight different tissues |
| --- | --- | --- |
| *tIL7c* | 52.58 | 49.11 |
| *tIL7-sv1* | 9.29 | 8.93 |
| *tIL7-sv2* | 12.37 | 10.71 |
| *tIL7-sv3* | 4.12 | 4.46 |
| *tIL7-sv4* | 2.06 | 3.57 |
| *tIL7-sv5* | 0.00 | 3.57 |
| *tIL7-sv6* | 0.00 | 2.68 |
| *tIL7-sv7* | 12.37 | 4.46 |
| *tIL7-sv8* | 5.15 | 10.71 |
| *tIL7-sv9* | 1.03 | 0.09 |
| *tIL7-sv10* | 1.03 | 0.09 |
